# Supplementary material for: PseudoknotVisualizer: Visualization of pseudoknots on three-dimensional RNA structures
Source: PLoS Comput Biol. 2025 Nov 20;21(11):e1013693. doi: 10.1371/journal.pcbi.1013693 (PMC12654949; doi:10.1371/journal.pcbi.1013693)
Supplement: S1 Text — DSSR v1.9.10-2020apr23; RNAView v2.0.0 (Jan 2024). (PDF) [file pcbi.1013693.s001.pdf]

S1. Base-pair detection methods (Base-pair annotators)

DSSR `v1.9.10-2020apr23`; RNAView `v2.0.0 (Jan 2024)`.
