# Supplementary material for: PseudoknotVisualizer: Visualization of pseudoknots on three-dimensional RNA structures
Source: PLoS Comput Biol. 2025 Nov 20;21(11):e1013693. doi: 10.1371/journal.pcbi.1013693 (PMC12654949; doi:10.1371/journal.pcbi.1013693)
Supplement: S2 Text — We render the drawing in layers. Given the allowed base pairs E, we perform greedy layering by a Nussinov-style dynamic programming pass that extracts a maximum-cardinality non-crossing subset at each layer. Each DP pass runs in O(n3) time and O(n2) space. This strategy is effective for visualization but does not guarantee the minimum number of layers. (PDF) [file pcbi.1013693.s002.pdf]

S2. Details of pseudoknot layer decomposition algorithm

We render the drawing in layers. Given the allowed base pairs $E \subseteq \{(i,j) \mid 1 \le i < j \le n\}$, each layer is obtained as a maximum-cardinality non-crossing subset $S \subseteq E$ computed by a Nussinov-style dynamic program (Algorithm 1) (1). We remove S from E and repeat until E is empty. Each DP pass runs in $O(n^3)$ time and $O(n^2)$ space. This greedy layering is effective for visualization but does not guarantee the minimum number of layers (2).

Algorithm 1 Greedy layering by Nussinov-style DP for non-crossing pairs

Require: sequence length n; allowed pairs $E \subseteq \{(i,j) \mid 1 \le i < j \le n\}$
Ensure: layers $\mathcal{L} = \left(S^{(1)}, S^{(2)}, \dots\right)$ where each $S^{(t)}$ is a maximum-cardinality non-crossing subset of the remaining edges

$\mathcal{L} \leftarrow [\,]$
while $E \neq \emptyset$ **do** ▷ Greedy layering
  initialize $M[1..n][0..n] \leftarrow 0$ and backpointers $P[1..n][0..n] \leftarrow$ None
  for $\ell = 1$ **to** n **do** ▷ fill by increasing span $j - i + 1 = \ell$
    for $i = 1$ **to** $n - \ell + 1$ **do**
      $j \leftarrow i + \ell - 1$
      $c_1 \leftarrow (M[i{+}1][j],\ \mathsf{skip\text{-}i})$
      $c_2 \leftarrow (M[i][j{-}1],\ \mathsf{skip\text{-}j})$
      $c_3 \leftarrow \left(M[i{+}1][j{-}1]{+}\mathbf{1}\{(i,j) \in E\},\ \mathsf{pair\text{-}ij}\right)$
      $c_4 \leftarrow \max_{i \le k < j} \left(M[i][k] + M[k{+}1][j],\ \mathsf{split\text{-}}k\right)$
      $(M[i][j], P[i][j]) \leftarrow \textsc{ArgMax}\{c_1, c_2, c_3, c_4\}$
  $S \leftarrow \textsc{Backtrack}(P, 1, n)$
  $\mathcal{L}.\textsc{append}(S)$
  $E \leftarrow E \setminus S$
return $\mathcal{L}$
function $\textsc{Backtrack}(P, i, j)$
  if $i \ge j$ **then**
    return $\emptyset$
  else if $P[i][j] = \mathsf{skip\text{-}i}$ **then**
    return $\textsc{Backtrack}(P, i{+}1, j)$
  else if $P[i][j] = \mathsf{skip\text{-}j}$ **then**
    return $\textsc{Backtrack}(P, i, j{-}1)$
  else if $P[i][j] = \mathsf{pair\text{-}ij}$ **then**
    return $\{(i,j)\} \cup \textsc{Backtrack}(P, i{+}1, j{-}1)$
  else ▷ split-k
    $k \leftarrow \mathsf{index}(P[i][j])$
    return $\textsc{Backtrack}(P, i, k) \cup \textsc{Backtrack}(P, k{+}1, j)$

Time/space: each DP pass runs in $O(n^3)$ time and $O(n^2)$ space; the greedy layering is effective for visualization but does not minimize the number of layers.

References

[1] Smit S, Rother K, Heringa J, Knight R. From knotted to nested RNA structures: a variety of computational methods for pseudoknot removal. RNA. 2008;14(3):410–416.

[2] Zok T, Badura J, Swat S, Figurski K, Popenda M, Antczak M. New

models and algorithms for RNA pseudoknot order assignment. Int J Appl Math Comput Sci. 2020;30(2):315–324.
