# Supplementary material for: PseudoknotVisualizer: Visualization of pseudoknots on three-dimensional RNA structures
Source: PLoS Comput Biol. 2025 Nov 20;21(11):e1013693. doi: 10.1371/journal.pcbi.1013693 (PMC12654949; doi:10.1371/journal.pcbi.1013693)
Supplement: S3 Text — Code: https://github.com/TakumiOtagaki/PseudoknotVisualizer (license MIT). The scripts to recreate the graphs are provided. The full chain list (PDB ID + chain) is available at analysis/dataset/ pdbid_chains.txt in the repository. (PDF) [file pcbi.1013693.s003.pdf]

S3. Data and Code Availability

Code: `https://github.com/TakumiOtagaki/PseudoknotVisualizer` (license `MIT`). The scripts to recreate the graphs are provided. The full chain list (PDB ID + chain) is provided at analysis/dataset/pdbid_chains.txt in the repository.
