# Supplementary material for: PseudoknotVisualizer: Visualization of pseudoknots on three-dimensional RNA structures
Source: PLoS Comput Biol. 2025 Nov 20;21(11):e1013693. doi: 10.1371/journal.pcbi.1013693 (PMC12654949; doi:10.1371/journal.pcbi.1013693)
Supplement: S1 Fig — (a) Distribution of the number of layers when using RNAView as the base-pair annotator. The trend is consistent with the DSSR-based results; including non-canonical base pairs increases the number of layers compared with using canonical pairs only. (b) Distribution of canonical and non-canonical base pairs across layers. Non-nested layers have a higher fraction of non-canonical base pairs than the core layer. (PDF) [file pcbi.1013693.s004.pdf]

S1 Fig. Result by RNAView

We replicated the analyses using RNAView as the base-pair annotator. The main trends are consistent with the DSSR-based results in Figure 3.

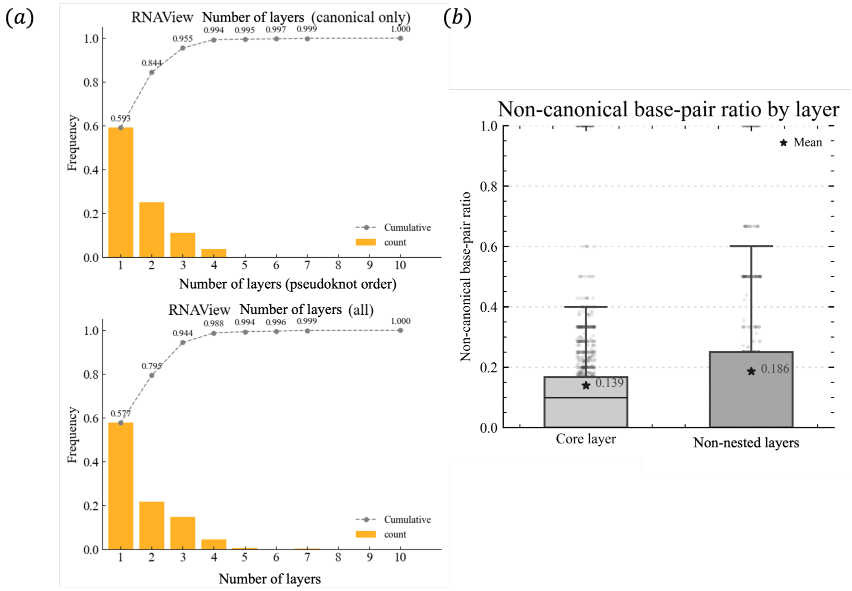

Figure 1: **Supplementary analyses.** (a) Distribution of the number of layers when using RNAView as the annotator. Consistent with the DSSR-based analysis in Fig. 3(a), including non-canonical base pairs in the decomposition increases the number of layers compared with using canonical pairs only. (b) Distribution of canonical and non-canonical base pairs across different decomposed layers in RNA molecules that contain at least one pseudoknot or other non-nested interactions. Similar to Fig. 3(b), non-nested layers exhibit a higher fraction of non-canonical base pairs than the core layer.
