# Supplementary material for: PseudoknotVisualizer: Visualization of pseudoknots on three-dimensional RNA structures
Source: PLoS Comput Biol. 2025 Nov 20;21(11):e1013693. doi: 10.1371/journal.pcbi.1013693 (PMC12654949; doi:10.1371/journal.pcbi.1013693)
Supplement: Attachment — Submitted filename: PLOS-PseudoknotVisualizer.pdf [file pcbi.1013693.s005.pdf]

The manuscript by Otagaki et al. presents PseudoknotVisualizer, a software tool designed to identify and visualize pseudoknots in RNA tertiary structures. The tool is available as an extension for PyMOL to enable interactive visualization of various layers of pseudoknots, and it also offers a Command Line Interface (CLI) for integration with both PyMOL and Chimera. A statistical analysis of pseudoknots within the Protein Data Bank (PDB) was also performed.

While reviewing the manuscript, I followed the installation and usage guidelines provided on GitHub. Below are my observations and suggestions regarding PseudoknotVisualizer.

Installation

PyMOL extension installation

There are gaps in the installation instructions that need to be addressed to ensure a seamless setup process for users.

1. Create and activate the `pymol` Conda environment, then clone the `PseudoknotVisualizer` GitHub repository. These two steps both work.
2. Clone the `RNAView` GitHub repository within the `PseudoknotVisualizer` project root directory. This detail should be made explicit. I initially installed `RNAView` directly under `$HOME` and set the `RNAVIEW` environment variable, but PseudoknotVisualizer could not locate it. Only after reviewing `config.py` (included below) did I identify where the issue was.
3. The installation also requires creating a folder named `intermediate` (as referenced below in `config.py`), but this detail was neither mentioned in the instructions nor included in the repository.

```
# excerpt from config.py
# ------------- Do not edit below this line. ------------------
 PseudoKnotVisualizer_DIR = Path(__file__).parent
 INTERMEDIATE_DIR = PseudoKnotVisualizer_DIR / "intermediate"
 # ------------------------------------------------------------

 # The variable RNAVIEW is the path to the RNAView directory and
RNAVIEW/bin/rnaview is the path to the RNAView executable.
 RNAVIEW = PseudoKnotVisualizer_DIR / "RNAView"
 RNAVIEW_PATH = RNAVIEW / "bin"
```

CLI installation

- The section 'Installation for CLI User' should be placed before the 'CLI Usage' section in the documentation.

- Step 2: `pip install -r requirements.txt` does not work because the file `requirements.txt` is not provided in the repository.
- Based on the error messages, I ran `conda install -c bioconda bio` to successfully get the CLI version running.

Overall, consolidating the installations of both the CLI and the PyMOL extension into a single Conda environment would streamline the setup process for users. Additionally, automating the installation through a script would significantly enhance convenience and ease of use.

The RNAView dependency

PseudoknotVisualizer relies on RNAView to extract base-pair information from atomic coordinates. However, it is important to note that RNAView is no longer actively maintained. In fact, the authors identified a buffer overflow bug in RNAView when the installation path exceeds 80 characters in length. This issue was reported in March 2025 but remains unresolved.

Notably, the underlying algorithm for identifying base pairs and calculating parameters in RNAView is based on an earlier version of 3DNA. Interestingly, traces of 3DNA can still be found within the RNAView codebase (see below). For further context, please refer to the thread 'Source distribution/Linux packaging' from a decade ago on the 3DNA Forum: http://forum.x3dna.org/index.php?topic=534.msg1876.

```
# Within the git repo clone folder of RNAView
❯ git grep -i 3dna
BASEPARS/Atomic_A.pdb:REMARK    DNA structure generated by the 3DNA package
BASEPARS/Atomic_C.pdb:REMARK    DNA structure generated by the 3DNA package
BASEPARS/Atomic_G.pdb:REMARK    DNA structure generated by the 3DNA package
BASEPARS/Atomic_I.pdb:REMARK    DNA structure generated by the 3DNA package
BASEPARS/Atomic_P.pdb:REMARK    DNA structure generated by the 3DNA package
BASEPARS/Atomic_T.pdb:REMARK    DNA structure generated by the 3DNA package
BASEPARS/Atomic_U.pdb:REMARK    DNA structure generated by the 3DNA package
src/analyze.c:/* calculate and print out 3DNA recommended local parameters */
src/fpair_sub.c:/* read in H-bond length upper limit etc from <misc_3dna.par>
*/
```

3DNA has been replaced by DSSR, which is actively maintained. Interestingly, DSSR is cited in the manuscript (**Reference #11**). Given that DSSR offers JSON output and clearly identifies canonical base pairs, integrating it into PseudoknotVisualizer would be straightforward.

Presentation of results

- In the PyMOL extension, PseudoknotVisualizer assigns distinct colors to different layers of pseudoknots for enhanced visualization. It would be helpful to put different pseudoknot layers

into separate selections in PyMOL so that each can be styled individually.

- PseudoknotVisualizer automatically renumbers residues to start from 1 'to avoid the error caused by non-sequential residue numbers in the input PDB file.' However, this approach is undesirable because preserving the original numbering is crucial. In PDB files, it is common for residue numbers to be non-sequential, start from a number other than 1, or even include repetitions with insertion codes. This limitation in PseudoknotVisualizer is partly due to its reliance on RNAView; however, integrating DSSR could address this issue effectively.
- In the section 'Statistics on Pseudoknots,' it is unclear how the authors obtained statistical data on the distribution of non-canonical and canonical base pairs across each pseudoknot layer since only canonical (WC and Wobble) pairs are considered in the identification of pseudoknots. To make the conclusion regarding the enrichment of non-canonical pairs in pseudoknot layers more convincing, a concrete example, such as 1EHZ (the yeast phenylalanine tRNA), would help clarify this point.

Some specific points to consider

- lines 24-25: "This software allows users to input RNA tertiary structure data in PDB format." PDB is a legacy format that is being phased out, so support for the mmCIF format should be added (or mentioned explicitly).
- Fig 2 legend: "white represents unpaired bases;" -- White represents non-canonical base pairs or unpaired bases. Again, having the different layers in separate PyMOL selections would facilitate further analysis.
- line 72: "Using the pseudoknot layer decomposition algorithm described in Section 2" -- section numbering error. The "Main Features" section is not numbered, and current section "Statistics on Pseudoknots" should be Section 3 (instead of 2).
- line 78: "a dataset of 1,788 RNA chains" -- Please specify these RNA chains long with their PDB IDs in the supplementary materials or the GitHub repository.
