# Supplementary material for: PseudoknotVisualizer: Visualization of pseudoknots on three-dimensional RNA structures
Source: PLoS Comput Biol. 2025 Nov 20;21(11):e1013693. doi: 10.1371/journal.pcbi.1013693 (PMC12654949; doi:10.1371/journal.pcbi.1013693)
Supplement: Attachment — Submitted filename: Response to Reviewers.pdf [file pcbi.1013693.s006.pdf]

Point-by-point responses

Reviewer #1:

> *The paper by Otagaki et al. presents PseudoknotVisualizer, a visualization plugin designed for PyMOL and Chimera that highlights pseudoknot-forming base pairs in RNA 3D structures using distinct colors. The tool integrates existing third-party algorithms to detect base pairs and identify pseudoknots of various orders, and then applies color coding to these elements within a rendered 3D model. While the plugin is of some practical use for visualizing RNA pseudoknots, the work appears to be primarily an engineering-level implementation effort, suitable for an undergraduate-level project.*
> *The core computational tasks - base pair annotation and pseudoknot classification - are delegated entirely to external tools, with the authors contributing the integration of these tools and visualization layer. Scientifically, the novel aspect of the paper lies in the descriptive statistics of canonical and noncanonical base pairs across different structural layers and pseudoknot orders.*

Acknowledgement to Reviewer1. We thank Reviewer 1 for a careful and constructive assessment. Your comments prompted us to clarify scope and terminology, document support for modern formats, justify algorithmic choices, and strengthen reproducibility and presentation. In response, we now make mmCIF support explicit and add DSSR as a maintained alternative annotator; we clarify our topological use of "pseudoknot" and position our contribution relative to RNApdbee; we describe the layered dynamic-programming procedure; and we re-ran all statistics on a curated RNAsolo dataset, correcting figures, captions, and axes accordingly. These changes substantially improve clarity, transparency, and practical utility of the work.

Notably, the plugin does not support the currently recommended mmCIF input format or new-style PDB identifiers, which significantly limits its applicability to the newest and large RNA structures. These are my main concerns regarding the manuscript. Additional comments are outlined below.

Response. Thank you for pointing this out. Our software has originally supported mmCIF, however, it was not stated in the previous manuscript. We now state it explicitly in the manuscript (p.2 l.37–39, p.2 l.49–p.3 l.52, Fig1 caption, p.3 l.69 and p.4 l.82).

To address this need, we developed the PseudoknotVisualizer, a tool that visualizes pseudoknots within RNA tertiary structures. This software allows users to input RNA tertiary structure data in the PDB or mmCIF format. It identifies pseudoknots and clarifies their spatial layouts in the widely used 3D molecular viewers, Chimera (12) and

modules; RNAView (10) and DSSR (11; 15; 16)) to extract base-pairing information from PDB or mmCIF files. Once RNAView or DSSR is installed, users can analyze an RNA object and a specified chain in PyMOL by entering `pkv RNAobject[, chainID, annotator]`. Since RNAView and DSSR adopt different geometric criteria and differ in how base-pairing interactions are handled, their outputs can legitimately diverge for the

Fig 1. Workflow of the PseudoknotVisualizer. The software offers two usage options: a PyMOL extension and a Command Line Interface (CLI). The left figure illustrates the PyMOL extension, where a tertiary structure loaded into PyMOL (in this case, 5TPY) is analyzed and visualized by entering `pkv 5TPY` in the command line. This command applies color to the pseudoknot layers, with red indicating pseudoknot layer 1 and blue indicating layer 2, while additional layers (if present) can be visualized using other colors. Note that the entire molecule was pre-colored white to highlight the red and blue pseudoknot layers. The right figure demonstrates the CLI workflow. Like the PyMOL extension, the CLI takes a PDB or mmCIF file as input and generates a script for coloring in Chimera or PyMOL. By pasting this script into the command line of the specified viewer, users can visualize pseudoknot layers by applying colors to the structure.

The CLI provides a method to use PseudoknotVisualizer without the need for a graphical user interface. By executing the following command, users can generate a coloring script for a specific chain within a PDB or mmCIF file.

```
python PseudoknotVisualizer/CLI_PseudoknotVisualizer.py \
  -i /path/to/your_pdb.pdb \ # Supports PDB and mmCIF formats.
  -o /path/to/output_script.txt \
  -a RNAView \ # or DSSR (annotator)
  -c chain_id \
  -f pymol
```

A brief explanation of how PseudoknotVisualizer operates is as follows: it begins by extracting base pair information from the user-provided 3D structural data using the external module RNAView or DSSR. Based on the complete list of base pairs, it identifies sets of pseudoknot-free base pairs, which are either mutually nested or exhibit bifurcation relationships between their components. Using dynamic programming,

(1) The Introduction opens with the sentence: "Pseudoknot structures are complex RNA formations where two or more groups of base pairs form between distinct loop regions of the other base pairing groups." First, the phrase "regions of the other base pairing groups" is unclear and should be rephrased for precision.
More importantly, this definition of pseudoknots appears to be overly narrow. Pseudoknots form when base-pairing interactions occur between two single-stranded regions separated by a stem, and these single-stranded regions do not necessarily have to be loops - they can include other unpaired segments such as extended single-stranded tails. If the authors define pseudoknots strictly as interactions between loops, does this imply that their software cannot handle cases where pseudoknots involve a loop and a non-loop single-stranded region? Clarification is needed both in the definition and in the scope of the tool's capabilities.

Response(1). We thank the reviewer for pointing this out. We agree that our original definition was unnecessarily narrow and potentially misleading. In the revision, we have rephrased the opening sentence along the suggested lines: *"Pseudoknots form when base-pairing interactions occur between two single-stranded regions separated by a stem."* We now also explicitly use the term *"non-nested"* to make the definition unambiguous. (p.2 l.1–3, l.8–11)

Our previous phrasing reflected our assumption that the "extended single-stranded tails" could be often referred to as the "exterior loop" in structure prediction studies (for example, the explanatory figure on the DSSR homepage: https://x3dna.org/articles/exterior-loop-in-rna-secondary-structure); however, we recognize that this assumption is not intuitive. To avoid the confusion, we have rephrased this sentence in the revised manuscript. We further clarify that throughout the manuscript we use "pseudoknot" in a broad, topological sense, consistent with prior literature. We appreciate this helpful comment, which improved the clarity and scope of our definition.

Pseudoknots are structural motifs formed by base pairing between two single-stranded regions separated by an intervening stem; their base pairs are non-nested (they cross when drawn in two dimensions). In many contexts, the term "pseudoknot" is restricted

such cases can also be viewed more generally as non-nested interactions. Throughout this work, unless otherwise specified, we use "pseudoknot" in a broad, topological sense to include both canonical and non-canonical cases. Here, "base pair" denotes any annotated nucleotide–nucleotide interaction (canonical or non-canonical) in the structure. These structures are integral to various RNA tertiary formations and play

(2) In the second paragraph of the Introduction, the authors state: "However, there are no useful tools that can efficiently identify and clarify pseudoknots in 3D RNA structure data." This statement is inaccurate and somewhat surprising, especially considering that the authors themselves cite RNApdbee, a well-established tool that directly addresses this task. RNApdbee analyzes RNA 3D structures (!), annotates base pairs including pseudoknots, and generates 2D structural visualizations in which pseudoknots of different orders are highlighted with distinct colors and symbols in an extended dot-bracket notation.
The tool allows users to choose among multiple algorithms (four for base-pair annotation and five for topological classification of pseudoknots) and supports modern data formats, including mmCIF. Although RNApdbee does not provide 3D visualization like PseudoknotVisualizer, it offers significantly broader functionality for the analysis, annotation, and topological decomposition of pseudoknots in RNA 3D structures. I recommend that the authors revise this claim to reflect the actual state of available tools and clarify how their approach complements or differs from existing solutions.

Response(2). We agree that our original wording “no useful tools” was inadequate. We now explicitly acknowledge **RNApdbee** and summarize its functionality—secondary-structure annotation and 2D representations using **multiple annotators and multiple decomposition algorithms** (p.2 l.25–31). We also clarify the originality of our work as a **complementary contribution**: an interactive **3D** (PyMOL-integrated) visualization of the **pseudoknotted (non-nested) interactions** (p.2 l.32–35). In addition, we explain that different choices of annotator and decomposition algorithms can lead to different results, and we have revised the text to make these points explicit, which is described in the response (3). As noted above, our tool supports mmCIF.

Several applications exist for analyzing pseudoknots: some can generate 2D diagrams of secondary structures with pseudoknots (7; 8; 9), while others identify pseudoknot formation based on the base-pairing information (10; 11). Among these softwares, RNApdbee (and its updated version RNApdbee 2.0) (8; 9) gives users a choice of base-pair annotators and multiple algorithms for the pseudoknot layer decomposition and presents their calls in a common format; users can obtain dot-bracket strings including pseudoknots for downstream analysis.

Nevertheless, seamless integration of pseudoknot-aware annotation with interactive 3D visualization within a single workflow remains limited. This can hinder rapid inspection of spatial context and long-range interactions when studying pseudoknots, which is important for RNA tertiary structure analysis, including prediction.

(3) The authors have arbitrarily selected specific tools for base-pair annotation (Yang et al., 2003) and pseudoknot topology decomposition (Smit et al., 2008), but they do not justify why these particular methods were chosen.
Are the authors aware that multiple algorithms exist for both base-pair detection and pseudoknot classification, and that these tools can yield substantially different results? In fact, the same 3D RNA structure can be interpreted as having very different maximum pseudoknot orders depending on the methods used. For example, in the case of PDB entry 3J7Q, the identified pseudoknot order may vary between 4, 5, 6, or even 7 when analyzed with different algorithms available in RNApdbee.
The current implementation of PseudoknotVisualizer relies on a fixed pipeline, using tools preselected by the authors, without informing the user that these choices affect the outcome or that alternative tools might lead to different interpretations. This lack of transparency is problematic, as it may mislead users into assuming that the presented results are deterministic and definitive. I strongly recommend that the authors either provide a rationale for their choices or, preferably, implement options for algorithm selection or include a warning regarding result variability.

Response(3).

We thank the reviewer for raising this important point.

<u>*Base-pair annotation.*</u>
Our initial implementation to **RNAView** out of convenience and familiarity, though there exist multiple annotators. In accordance with the **Reviewer #3**'s suggestion, we have added DSSR as an optional base-pair annotation. Now, users can select either RNAView or DSSR. We have also explicitly stated that the combination of base-pair annotation methods and pseudoknot decomposition algorithms could lead to different results (p.3 l.53-57, p.4 l.122-p.5 l.124, Overview section in github). In addition, we state our plan to integrate other base-pair annotators; this is mentioned in Section 4 (Availability and Future Directions; p.6 l.149-152).

<u>*Pseudoknot topology decomposition.*</u>
As the reviewer pointed out, alternative algorithms may yield different results. We now clearly state it in the manuscript (p.3 l.53-57, p.4 l.106-107), and we have added a forward-looking note in Section 4 (Availability and Future Directions; p.6 l.152-155). We explained the reason why we chose the algorithm. We summarize the reason as follows; the greedy algorithm maximizes the number of base pairs in the core layer and minimizes that in pseudoknot layers, which clarifies the pseudoknot visualization. The approach is also intuitive and validated by its use in CaCoFold (Rivas E., 2020).

`annotator]`. Since RNAView and DSSR adopt different geometric criteria and differ in how base-pairing interactions are handled, their outputs can legitimately diverge for the same input. At the same time, multiple strategies exist for pseudoknot-layer decomposition, and different choices can lead to different decompositions (i.e., different layerings). Each layer is colored differently to enhance visual distinction, making it

While we selected this strategy for these advantages, we note that alternative algorithms could yield different results.

RNAView, 48,306 canonical and 6,509 non-canonical pairs. These differences reflect distinct geometric criteria and treatment of nucleotide interactions; however, the key

trends reported below were consistent across both annotators.

> *(4) At the end of the Introduction, the authors write that their script might enhance researchers' intuitive understanding of pseudoknots within RNA structures and to accelerate developments in RNA tertiary structure prediction methods by providing clear and immediate visualization. The authors may also consider acknowledging the current limitations in RNA secondary structure prediction when pseudoknots are involved. While several algorithms exist that attempt to predict secondary structures including pseudoknots, the accuracy of such predictions remains low. This limitation is well illustrated in recent analyses, such as the study by Justyna et al. (2023), which highlights the challenges associated with reliable prediction of RNA secondary structures with and without pseudoknots.*

Response(4).

We thank the reviewer for this helpful suggestion. We have stated that accurate prediction of RNA structures containing pseudoknots remains challenging at the end of the Introduction (p.2 l.42–45). In particular, secondary structure prediction methods that include pseudoknots still show low accuracy, as highlighted by Justyna et al. (2023). By making this point explicit, we clarify that our contribution is not a prediction algorithm but rather a visualization and analysis tool that complements existing methods.

structure prediction methods by providing clear and immediate visualization. At the same time, accurate RNA secondary-structure prediction—particularly in the presence of pseudoknots—remains difficult; recent benchmark analyses across Machine Learning (ML) and non-ML methods report variable, often modest accuracy (14).

(5) "Consequently, the distinct sets of base pairs identified in this manner are inherently related as pseudoknots. These sets are then sequentially assigned to layers, starting with layer0 for the largest ..." - regarding this fragment, it would be helpful to clarify explicitly that layer0 refers to the core structure, i.e., the base-pairing layer that does not contain any pseudoknots. While this may be implied, stating it directly would improve clarity for readers who are less familiar with pseudoknot decomposition convention used in this work.

Response(5).

Thank you for the clear suggestion to use "core structure." We agree that this phrasing improves readability. To keep the terminology consistent with our use of "pseudoknot layer," however, we have rephrased "layer0" to "core layer" (p.4 l.91–94).

related as pseudoknots. These sets are then sequentially assigned to layers, starting with layer0 (core layer) for the largest set, followed by layer1, and so on (pseudoknot layer), ensuring a clear hierarchical representation of the base pair sets. The number of

(6) The sentence "Using the pseudoknot layer decomposition algorithm described in Section 2" appears to refer to a description of the algorithm that is actually presented in the ***unnumbered section titled Main Features****, which either functions as part of the Introduction or was unintentionally left without a section number. In the current structure of the manuscript, Section 2 contains statistical analyses rather than a description of the decomposition algorithm. I recommend correcting this reference to avoid confusion and to ensure accurate navigation for the reader.*

Response(6). Thank you for pointing this out. We have corrected the section numbering and the in-text cross-reference.

2 Design and Implementation

(7) "After excluding complexes with proteins or other molecules for simplicity and selecting only structures composed solely of RNA, we obtained a dataset of 1,788 RNA chains" - Since the content of the PDB is constantly updated, I recommend that the authors specify the exact date of data retrieval to ensure reproducibility. As an alternative source for curated RNA-only 3D structures, the authors may also consider referring to the RNAsolo database (https://rnasolo.cs.put.poznan.pl/), which facilitates the extraction of RNA chains not involved in complexes with proteins or ligands.

Response(7). We appreciate the reviewer for suggesting reproducibility. In accordance with the reviewer's suggestion, we **re-ran the analysis using RNAsolo (v2) as our primary data source** for curated RNA-only structures. The dataset was **retrieved on July 25, 2025**, and we now report this retrieval date explicitly (p.4 l.109–113). We also document the exact filters used, including a resolution cutoff of ≤ 4.0 Å. With RNAsolo, the downstream analyses have also been updated in the revised manuscript.

Using the pseudoknot layer decomposition algorithm described in Section 2, we decomposed 1,915 chains from RNAsolo (22) into pseudoknot-free layers (filters: repository=BGSU, redundancy=all class members, molecule=solo RNA, method=all, resolution ≤ 4.0, Å; accessed 2025-07-25). RNAsolo provides pure RNA structures, deriving from Protein Data Bank. Canonical base pairs were defined as Watson–Crick pairs (A–U, G–C) and wobble pairs (G–U) (23). While our default visualizations used canonical pairs, we also report analyses that include non-canonical pairs. Among these 1,915 chains, after excluding entries with no base pairs/layers, the analyzable set sizes were: RNAView (canonical only) 1,726 chains, of which 703 had at least one pseudoknot layer; RNAView (all pairs) 1,789 chains, of which 756 had at least one pseudoknot layer; DSSR (canonical only) 1,718 chains, of which 687 had at least one pseudoknot layer; DSSR (all pairs) 1,727 chains, of which 788 had at least one pseudoknot layer. Using DSSR, we obtained 44,698 canonical and 8,331 non-canonical base pairs; using RNAView, 48,306 canonical and 6,509 non-canonical pairs. These differences reflect distinct geometric criteria and treatment of nucleotide interactions; however, the key trends reported below were consistent across both annotators.

Figure 3a shows the distribution of pseudoknot order (i.e., the number of layers under our decomposition), annotating with DSSR. With canonical pairs only, most chains have ≤ 3 layers; including non-canonical pairs slightly shifts the tail, but orders > 4 remain rare. The observed distribution of pseudoknot-free pseudoknot orders is consistent with findings from previous studies (8).

We then extracted chains which have at least one pseudoknot layer. Figure 3(b) summarizes, for each layer, the per-chain fraction of non-canonical versus canonical pairs. The layer0 (core layer) is dominated by canonical pairs, whereas pseudoknot layers show a higher non-canonical fraction. The corresponding RNAView results show the same trend (Supplementary Figure S1).

Using the pseudoknot layer decomposition algorithm described in Section 2, we decomposed 1,915 chains from RNAsolo (22) into pseudoknot-free layers (filters: repository=BGSU, redundancy=all class members, molecule=solo RNA, method=all, resolution ≤ 4.0, Å; accessed 2025-07-25). RNAsolo provides pure RNA structures,

> (8) In the sentence "These 1,355 chains contained a total of 24,047 canonical base pairs and 7,198 non-canonical base pairs" it is unclear whether these statistics refer to the entire structures or only to the regions involved in pseudoknot formation. I recommend that the authors clarify this point in the manuscript.

Response(8).

In accordance with the reviewer's suggestion, we now state clearly what each panel summarizes: in **Fig. 3(a)** derived from entire structures using RNAsolo; **Fig. 3(b)** derived from the structures that contain at least one pseudoknot (p.5 l.131-138, p.7 Fig 3.). Since we re-ran the analysis using RNAsolo, the results have changed. We also replaced the earlier mean-only pie chart with box plots to convey distributional information (p.7 Fig 3).

(9) In the caption of Figure 3, the phrase "number of layer" should be corrected to "number of layers".

Response(9). Thank you for pointing this out. We corrected the phrase to **"number of layers."** (p.7 Fig 3.)

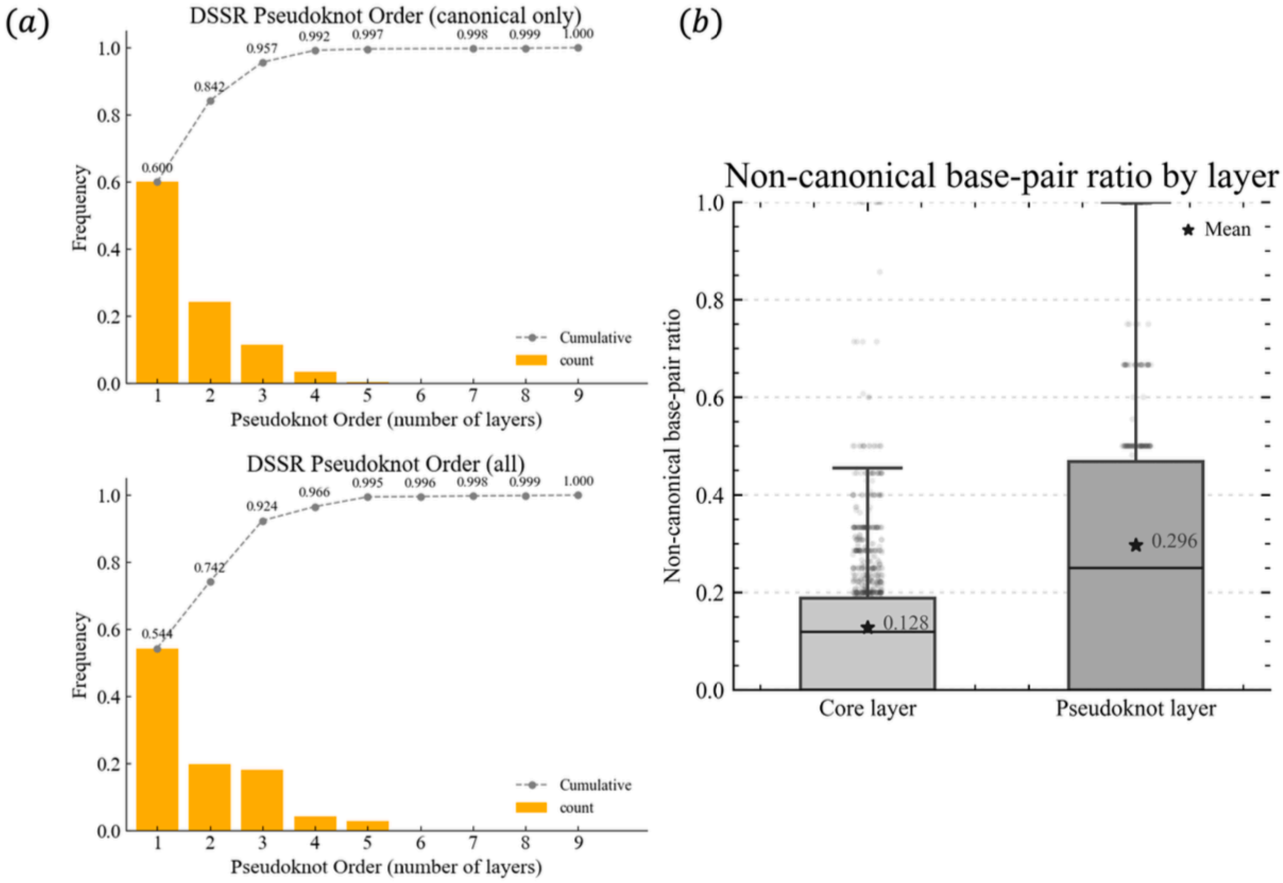

(10) In Figure 3(b), the two adjacent plots use different vertical axis scales: the left plot ranges from 0 to 0.5, while the right plot ranges from 0 to 0.35. Using inconsistent y-axis scales in side-by-side plots is a classic form of statistical misrepresentation and makes it difficult to accurately compare the data between the two charts. I strongly recommend adjusting the figure so that both plots use the same y-axis scale to enable proper visual comparison.

Response(10). We agree that inconsistent y-axis leads to misunderstandings. In accordance with the reviewer's recommendation, we have used the same y-axis scale across the plots in Fig. 3(a) (previous Fig. 3(b)).

Reviewer #2:

>*This manuscript introduces a visualization tool to directly identify non-nested RNA base pairs within 3D structural renderings of all atoms of an RNA structure, such as PyMOL.*

Acknowledgement to Reviewer2. We are grateful to Reviewer 2 for the insightful guidance on terminology and interpretation. Your feedback helped us make our usage explicit—treating "pseudoknot" in a broad, topological sense while distinguishing canonical versus non-canonical cases in both text and analyses—and to align language with community expectations. We revised the abstract and title, added an explicit description of the layered DP approach and its relationship to prior work (e.g., CaCoFold), and reported results in paired views (canonical-only and all pairs) to avoid ambiguity. These revisions clarify what is being visualized, how layers are constructed, and how the statistics should be interpreted.

>*My main point about this manuscript is about the use of the word pseudoknot. I believe, they should be called non-nested interactions. Pseudoknots are a particular subset of non-tested interactions that involve Watson-Crick base pairing only. The rest of the non-nested interactions involve non-WC base pairing.*

>*The distintion is important, because pseudoknots consist of nested WC base pairs the stack forming helices are subject to the same evolutionary presures than the nested WC helix and also display covariation when homologous pseudoknots are aligned. The same is mostly not true for the rest of non-nested non-WC base pair interactions, which do not form helices. For instance this definition in the paper, "Pseudoknot structures are complex RNA formations where two or more groups of base pairs form between distinct loop regions of the other base pairing groups." should be replaced byt "two or more groups of WC base pairs". And overal the non-nested interactions includes pseudoknots and all other non-WC base pairs.*

Response.

We thank the reviewer for raising this important issue. We now realize the importance of the difference between canonical and non-canonical non-nested structures from an evolutionary perspective. In the revised manuscript we make our usage explicit at the outset. We introduced *canonical pseudoknots* (conventional pseudoknot; formed by Watson–Crick and wobble pairs) and *non-canonical pseudoknots* (involving at least one non-canonical pair). In this manuscript, we then defined *pseudoknots* in a broad, topological sense as **non-nested base-pairing interactions** between two single-stranded regions separated by a stem, including canonical and non-canonical pseudoknots (p.2 11-12). Although we understand that the definition is different from the conventional one, we believe that readers are unlikely to be confused.

Pseudoknots are structural motifs formed by base pairing between two single-stranded regions separated by an intervening stem; their base pairs are non-nested (they cross when drawn in two dimensions). In many contexts, the term "pseudoknot" is restricted to interactions formed by canonical base pairs (Watson–Crick and wobble), which we refer to here as canonical pseudoknots. In contrast, when a pseudoknot involves at least one non-canonical base pair, we refer to it as a non-canonical pseudoknot—although such cases can also be viewed more generally as non-nested interactions. Throughout this work, unless otherwise specified, we use "pseudoknot" in a broad, topological sense to include both canonical and non-canonical cases. Here, "base pair" denotes any annotated nucleotide–nucleotide interaction (canonical or non-canonical) in the structure. These structures are integral to various RNA tertiary formations and play

>Also the abstract statement
>"canonical base pairs predominate in the main layer (78.6 %), while pseudoknot layers are enriched in non-canonical pairs (50.4 %);"
>should be re-written as "non nested layers include about ~50% pseudoknots versus other non-canonical pairs".

Response.

Thank you for the suggestion. After re-running all analyses on **RNAsolo (v2)**—a curated dataset of pure RNA chains—we found that the **fraction of non-canonical base pairs within pseudoknot layers is ~30%**, not ~50%. We updated the abstract and Results accordingly. (p.1 Author Summary, p.4-5 Results section) We also note that absolute percentages can vary with the annotation engine; in the main text we use **DSSR**, and we provide **RNAView** results in the Supplement, which show the same trend.

To avoid terminology mismatches, we now make the mapping explicit in the Introduction: the reviewer's **"non-nested layers"** correspond to what we call **"pseudoknot layers,"** and our previous **"main layer"** is now **"core layer (layer 0)"**. Throughout the paper, "pseudoknot" is used in a **topological** sense (any non-nested interaction, regardless of pair type). Following this usage, we report the **fraction of non-canonical base pairs** in the pseudoknot layers, rather than contrasting "pseudoknots vs other non-canonical pairs," which could mix definitions.

our tool to 1,915 RNA chains from the PDB revealed that canonical base pairs predominate in the core layer (87 %), while pseudoknot layers are enriched in non-canonical pairs (30 %); most chains exhibit no more than three layers of

> it would be interesting to see what kind of nested base pair they are seeing in the main layer that are not WC that constitute more than 20% of them.

Response. Thank you for suggesting the interesting direction of the study. In this study, our focus is on a **visualization workflow** and the **layered representation** itself, rather than a fine-grained taxonomy of base-pair families. We therefore report aggregate proportions but intentionally **do not drill down** into the family-level composition of **non-WC pairs in the core layer (layer 0)**. A rigorous analysis would require a dedicated study that controls for the **annotator's taxonomy** (e.g., Leontis–Westhof classes), dataset composition, and resolution thresholds. We view this as valuable **future work** and plan to pursue it separately (p.5 l.141–146).

layers. Our next step is to conduct a comprehensive statistical analysis of these interactions to better understand RNA structure. Specifically, we will quantify the distribution of the type of non-canonical base-pairs across pseudoknot layers and structural contexts and assess their contributions to 3D structure folding. Insights from

--- The title should also be re-written.

Response. Thank you for pointing this out. We revisited the title and agreed that the lead-in phrase **“Enabling Direct”** was unnecessary. We removed it to make the title more concise and descriptive (p.1 title).

> -- *Also, the dp algorithm to select layers is not described. Also notice that using a layered DP to select maximal subsets of nested interactions has been used extensively before (example, CaCoFold).*

Response. We agree that the **layer-selection algorithm** needs to be described. We have added a concise, self-contained presentation of the **layered dynamic-programming (DP) procedure** used to extract, per layer, a **maximum-cardinality non-crossing subset** of interactions (p.4 l.94–107). The Supplement now includes the algorithm (pseudocode), a short correctness rationale, and the computational complexity (Supplement S2, Algorithm 1).

We also now **cite CaCoFold** as an example of prior work employing layered DP ideas, emphasizing that our contribution is a **visualization/interaction workflow** integrated with PyMOL rather than a novel decomposition algorithm.

these layers is called pseudoknot order (18). Further algorithmic details are provided in Supplementary Section S2. As related work, similar layered dynamic programming strategies have been used in RNA structure prediction, notably CaCoFold, which first derives a maximally nested structure under covariation-based positive/negative constraints and then incorporates remaining (potentially non-nested) interactions in subsequent layers (19). Our layering instead operates on base pairs annotated from 3D coordinates and optimizes the size of a non-crossing subset for visualization. We chose this greedy algorithm, which maximizes the number of base pairs at each step. In practice, this naturally maximizes the number of base pairs in the core layer, thereby minimizing those assigned to the pseudoknot layers. This, in turn, helps users to visually understand the spatial distribution of pseudoknots. In addition, the algorithm is not only intuitive but also supported by its successful application in CaCoFold (19). While we selected this strategy for these advantages, we note that alternative algorithms could yield different results.

S2. Algorithmic Details

We render the drawing in layers. Given the allowed base pairs $E \subseteq \{(i,j) \mid 1 \le i < j \le n\}$, each layer is obtained as a maximum-cardinality non-crossing subset $S \subseteq E$ computed by a Nussinov-style dynamic program (Algorithm 1) (17). We remove S from E and repeat until E is empty. Each DP pass runs in $O(n^3)$ time and $O(n^2)$ space. This greedy layering is effective for visualization but does not guarantee the minimum number of layers (18).

Algorithm 1 Greedy layering by Nussinov-style DP for non-crossing pairs

Require: sequence length n; allowed pairs $E \subseteq \{(i,j) \mid 1 \le i < j \le n\}$
Ensure: layers $\mathcal{L} = (S^{(1)}, S^{(2)}, \dots)$ where each $S^{(t)}$ is a maximum-cardinality non-crossing subset of the remaining edges

```
L ← [ ]
while E ≠ ∅ do                                         ▷ Greedy layering
    initialize M[1..n][0..n] ← 0 and backpointers P[1..n][0..n] ← None
    for ℓ = 1 to n do                    ▷ fill by increasing span j − i + 1 = ℓ
        for i = 1 to n − ℓ + 1 do
            j ← i + ℓ − 1
            c1 ← (M[i+1][j], skip-i)
            c2 ← (M[i][j−1], skip-j)
            c3 ← (M[i+1][j−1]+1{(i,j) ∈ E}, pair-ij)
            c4 ← max_{i≤k<j} (M[i][k] + M[k+1][j], split-k)
            (M[i][j], P[i][j]) ← ArgMax{c1, c2, c3, c4}
    S ← Backtrack(P, 1, n)
    L.append(S)
    E ← E \ S
return L
function Backtrack(P, i, j)
    if i ≥ j then
        return ∅
    else if P[i][j] = skip-i then
        return Backtrack(P, i+1, j)
    else if P[i][j] = skip-j then
        return Backtrack(P, i, j−1)
    else if P[i][j] = pair-ij then
        return {(i,j)} ∪ Backtrack(P, i+1, j−1)
    else                                                      ▷ split-k
        k ← index(P[i][j])
        return Backtrack(P, i, k) ∪ Backtrack(P, k+1, j)
```

Time/space: each DP pass runs in $O(n^3)$ time and $O(n^2)$ space; the greedy layering is effective for visualization but does not minimize the number of layers.

Reviewer #3:

> *The manuscript by Otagaki et al. presents PseudoknotVisualizer, a software tool designed to identify and visualize pseudoknots in RNA tertiary structures. The tool is available as an extension for PyMOL to enable interactive visualization of various layers of pseudoknots, and it also offers a Command Line Interface (CLI) for integration with both PyMOL and Chimera. A statistical analysis of pseudoknots within the Protein Data Bank (PDB) was also performed.*

Acknowledgement to Reviewer3. We sincerely thank Reviewer 3 for hands-on testing and detailed, practical suggestions that greatly improved robustness and usability. Acting on your comments, we reorganized and simplified installation (single conda environment, corrected documentation order, added requirements.txt, clarified RNAView path and intermediate/), integrated DSSR and its JSON output to preserve original residue numbering, created per-layer PyMOL selections, clarified how per-layer statistics are computed with the example(1EHZ) suggested by the reviewer, corrected figure legends, stated mmCIF support explicitly, and provided a complete chain list with retrieval date and filters. These changes make the software easier to install and use, and they enhance the manuscript's transparency and reproducibility.

>*While reviewing the manuscript, I followed the installation and usage guidelines provided on GitHub. Below are my observations and suggestions regarding PseudoknotVisualizer.*

>*# Installation*

>*## PyMOL extension installation*

>*There are gaps in the installation instructions that need to be addressed to ensure a seamless setup process for users.*

1. Create and activate the pymol Conda environment, then clone the PseudoknotVisualizer GitHub repository. These two steps both work.

*2. Clone the RNAView GitHub repository within the PseudoknotVisualizer project root directory. **This detail should be made explicit.***
I initially installed RNAView directly under $HOME and set the RNAVIEW environment variable, but PseudoknotVisualizer could not locate it. Only after reviewing config.py (included below) did I identify where the issue was.

```
# excerpt from config.py
# ------------ Do not edit below this line. -----------------
PseudoKnotVisualizer_DIR = Path(__file__).parent
INTERMEDIATE_DIR = PseudoKnotVisualizer_DIR / "intermediate"
# ---------------------------------------------------------
## The variable RNAVIEW is the path to the RNAView directory and
RNAVIEW/bin/rnaview is the path to the RNAView executable.
RNAVIEW = PseudoKnotVisualizer_DIR / "RNAView"
RNAVIEW_PATH = RNAVIEW / "bin"
```

Response (Installation—PyMOL extension / RNAView path). Thank you for flagging the path-resolution issue. Our code assumes, by default, that **RNAView** resides under PseudoknotVisualizer/RNAView, which was not made explicit. We have expanded the **README** to (i) state this default clearly and (ii) document two supported setups:

1. **Recommended (default):** clone RNAView directly into PseudoknotVisualizer/RNAView; or

2. **External install:** keep RNAView elsewhere and **update config.py (RNAVIEW and RNAVIEW_PATH).**

This should prevent the confusion you encountered when an external install was not being detected.

We added a dedicated subsection to **README.md** with step-by-step instructions and examples for both the in-repo and external RNAView layouts, including the exact variables to edit in config.py or the symlink command.

```python
# config.py (snippet)
from pathlib import Path

# ---------------- RNAView configuration ----------------
# - RNAVIEW_DIR: Directory that contains the RNAView installation.
#   If you installed RNAView elsewhere, set it like: RNAVIEW_DIR = Path("/opt/RNAView")
RNAVIEW_DIR = PseudoKnotVisualizer_DIR / "RNAView"

# - RNAVIEW_EXEC: Path to the RNAView binary.
#   Example (custom path): RNAVIEW_EXEC = Path("/opt/RNAView/bin/rnaview")
RNAVIEW_EXEC = RNAVIEW_DIR / "bin/rnaview"
# -------------------------------------------------------
```

> *3. The installation also requires creating a folder named intermediate (as referenced below in config.py), but this detail was neither mentioned in the instructions nor included in the repository.*

Response (Installation—intermediate/ directory). Agreed. The need for an intermediate/ folder was implicit in config.py but not enforced by the repository layout. We added an **empty .gitkeep** file under intermediate/ so the directory is present immediately after cloning. The README now also mentions that this folder is used for temporary artifacts.

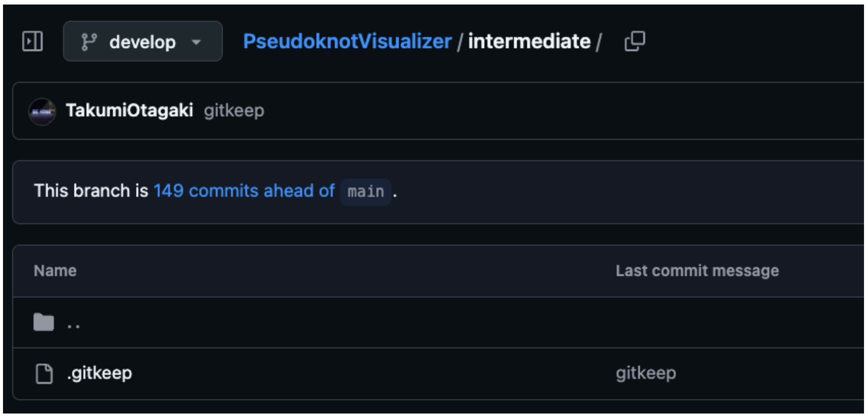

> *> ## CLI installation*
>
> *> The section 'Installation for CLI User' should be placed before the 'CLI Usage' section in the documentation. Overall, consolidating the installations of both the CLI and the PyMOL extension into a single Conda environment would streamline the setup process for users. Additionally, automating the installation through a script would significantly enhance convenience and ease of use.*

Response (CLI installation & docs order). We agree that the documentation order and environment setup could be streamlined. We have **moved "Installation for CLI Users" before "CLI Usage"** and **consolidated the CLI and PyMOL extension into a single Conda environment**. We also simplified the note about pymolrc.py: when installing under $HOME/PseudoknotVisualizer, users can now **copy–paste a short block**; for non-default locations, only the path needs to be edited (as before).

- Reordered the documentation so **installation precedes usage** for the CLI.
- Provided a **single Conda workflow** for both the CLI and PyMOL extension.

❖ Shortened the pymolrc.py instructions with a **ready-to-copy snippet** for the default $HOME layout.

Quickstart

```
# 1) Create env and install PyMOL (open-source build), which will be used for both GUI and CLI
conda create -n pkv python=3.11 -y
conda activate pkv
conda install -c conda-forge pymol-open-source -y

# 2) Clone repo + Python deps
cd ~ # optional but recommended for shorter paths (helps RNAView)
git clone https://github.com/TakumiOtagaki/PseudoknotVisualizer.git
cd PseudoknotVisualizer
pip install -r requirements.txt
# (Optional) Option B: DSSR — download binary to DSSR/x3dna-dssr (see below)

# 3) Install an annotator
# Option A: RNAView (build from source). If you want to use existing installation, see below.
git clone https://github.com/rcsb/RNAView.git
cd RNAView
make # if the name of the installation directory is long, you need to modify the RNAView scrip
ls bin # the binary rnaview will be found.
cd .. # back to PseudoknotVisualizer

# 4) Load the PyMOL extension automatically
cp pymolrc_template.py ~/.pymolrc_pkv.py

# 5) Launch PyMOL from the same env
conda activate pkv
pymol
```

After this step, you can use the PyMOL extension as described below at the PyMOL command line:

```
fetch 1ehz
pkv 1ehz
pkv 1ehz, include_all=1
```

If you prefer DSSR for annotation, install it and set `annotator=DSSR` (see below).

> *Step 2: pip install -r requirements.txt does not work because the file requirements.txt is not provided in the repository. Based on the error messages, I ran conda install -c bioconda bio to successfully get the CLI version running.*

Response (requirements). Thank you for catching this. We added a **requirements.txt** and re-ran the installation **from scratch** to verify that pip install -r requirements.txt works as documented

> *# The RNAView dependency*

> *PseudoknotVisualizer relies on RNAView to extract base-pair information from atomic coordinates. However, it is important to note that RNAView is no longer actively maintained. In fact, the authors identified a buffer overflow bug in RNAView when the installation path exceeds 80 characters in length. This issue was reported in March 2025 but remains unresolved. Notably, the underlying algorithm for identifying base pairs and calculating parameters in RNAView is based on an earlier version of 3DNA. Interestingly, traces of 3DNA can still be found within the RNAView codebase (see below). For further context, please refer to the thread 'Sourcedistribution/Linux packaging' from a decade ago on the 3DNA Forum: http://forum.x3dna.org/index.php?topic=534.msg1876.*

```
# Within the git repo clone folder of RNAView
❯ git grep -i 3dna
BASEPARS/Atomic_A.pdb:REMARK DNA structure generated by the 3DNA package
BASEPARS/Atomic_C.pdb:REMARK DNA structure generated by the 3DNA package
BASEPARS/Atomic_G.pdb:REMARK DNA structure generated by the 3DNA package
BASEPARS/Atomic_I.pdb:REMARK DNA structure generated by the 3DNA package
BASEPARS/Atomic_P.pdb:REMARK DNA structure generated by the 3DNA package
BASEPARS/Atomic_T.pdb:REMARK DNA structure generated by the 3DNA package
BASEPARS/Atomic_U.pdb:REMARK DNA structure generated by the 3DNA package
src/analyze.c:/* calculate and print out 3DNA recommended local parameters */
src/fpair_sub.c:/* read in H-bond length upper limit etc from <misc_3dna.par>
*/
```

> *3DNA has been replaced by DSSR, which is actively maintained. Interestingly, DSSR is cited in the manuscript (Reference #11). Given that DSSR offers JSON output and clearly identifies canonical base pairs, integrating it into PseudoknotVisualizer would be straightforward.*

Response. We appreciate the detailed background on **RNAView** and the suggestion to incorporate **DSSR**. We have now **implemented DSSR as an alternative annotator** (JSON-based), while keeping RNAView available for users who prefer it. We also **document the RNAView path and known caveats** in the README so users are not tripped up by layout/path-length issues.

- We added **DSSR** as a selectable annotator; updated the code and documentation accordingly (p.3 l.50–55 and README.md).
- **We recomputed statistics with DSSR**; **Fig. 3** (p.7) now reflects DSSR-based results, with an **RNAView replication in the Supplement** (Supplementary S4).
- We expanded the README.md: clarifications on RNAView location, and a note on potential path-related pitfalls with RNAView.

modules; RNAView (10) and DSSR (11; 15; 16)) to extract base-pairing information from PDB or mmCIF files. Once RNAView or DSSR is installed, users can analyze an RNA object and a specified chain in PyMOL by entering `pkv RNAobject[, chainID, annotator]`. Since RNAView and DSSR adopt different geometric criteria and differ in how base-pairing interactions are handled, their outputs can legitimately diverge for the same input. At the same time, multiple strategies exist for pseudoknot-layer

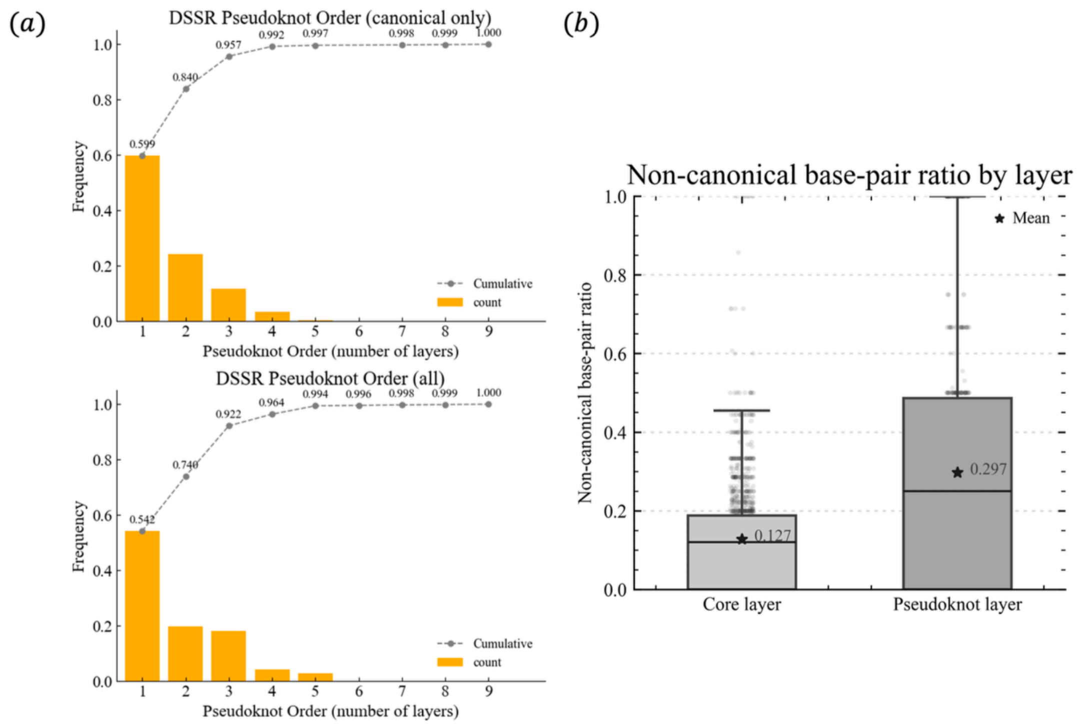

Fig 3. (a) Both graphs represent the frequency of pseudoknot order (number of pseudoknot-free layers) in RNA chains from PDB entries on the condition that these entries contain at least one base pair. The top graph considers only canonical base pairs, showing that over 80 % of chains have at most 2 layers. The bottom graph includes all base pairs, including non-canonical ones, demonstrating higher pseudoknot orders compared to the top graph. Nevertheless, over 80 % of chains still have at most 3 layers. (b) Distribution of canonical and non-canonical base pairs across different decomposed layers in RNA molecules that contain at least one pseudoknot, as determined by the PseudoknotVisualizer. Results shown were obtained using DSSR as the base-pair annotator.

```
# config.py (snippet)
from pathlib import Path

# ---------------- RNAView configuration ----------------
# - RNAVIEW_DIR: Directory that contains the RNAView installation.
#   If you installed RNAView elsewhere, set it like: RNAVIEW_DIR = Path("/opt/RNAView")
RNAVIEW_DIR = PseudoKnotVisualizer_DIR / "RNAView"

# - RNAVIEW_EXEC: Path to the RNAView binary.
#   Example (custom path): RNAVIEW_EXEC = Path("/opt/RNAView/bin/rnaview")
RNAVIEW_EXEC = RNAVIEW_DIR / "bin/rnaview"
# -----------------------------------------------------
```

> # Presentation of results

> - In the PyMOL extension, PseudoknotVisualizer assigns distinct colors to different layers of pseudoknots for enhanced visualization. It would be helpful to put different pseudoknot layers into separate selections in PyMOL so that each can be styled individually.

Response — Presentation of results (PyMOL selections). We agree that separating layers into distinct **PyMOL selections** improves usability. We implemented this feature so that each **pseudoknot layer** (and the **core layer**) is created as its own selection (p.3 l.62-64).

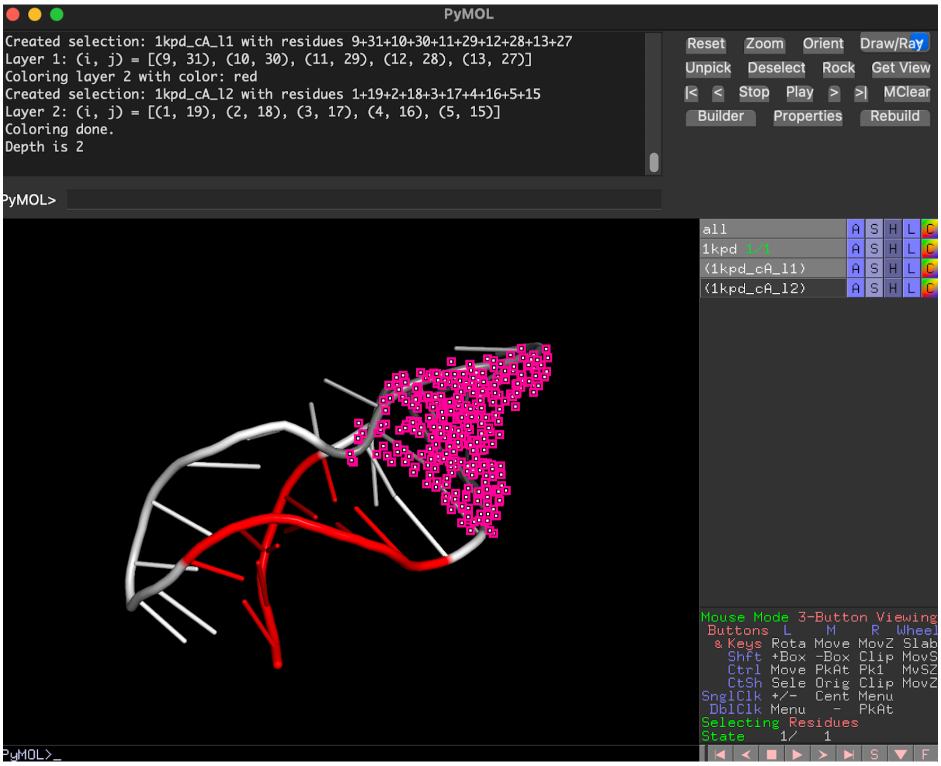

demonstrated in figure 2. In addition to coloring, PyMOL extension creates separate PyMOL selections for the different layers, which enables users to interactively analyze pseudoknots.

> *> - PseudoknotVisualizer automatically renumbers residues to start from 1 'to avoid the error caused by non-sequential residue numbers in the input PDB file.' However, this approach is undesirable because preserving the original numbering is crucial. In PDB files, it is common for residue numbers to be non-sequential, start from a number other than 1, or even include repetitions with insertion codes. This limitation in PseudoknotVisualizer is partly due to its reliance on RNAView; however, integrating DSSR could address this issue effectively.*

Response. We share the concern about re-numbering residues. Our earlier workaround (renumbering to start at 1) was motivated by **RNAView** limitations with non-sequential numbering. To address this properly, we **integrated DSSR** and now use its **JSON output** to preserve the **original residue identifiers** (including insertion codes) and avoid the numbering errors. For RNAView, we keep the legacy fallback documented, but **DSSR is the recommended annotator** when original numbering must be retained. We now clearly state it at the end of the README.md.

> *> - In the section 'Statistics on Pseudoknots,' it is unclear how the authors obtained statistical data on the distribution of non-canonical and canonical base pairs across each pseudoknot layer since only canonical (WC and Wobble) pairs are considered in the identification of pseudoknots. To make the conclusion regarding the enrichment of non-canonical pairs in*

pseudoknot layers more convincing, a concrete example, such as 1EHZ (the yeast phenylalanine tRNA), would help clarify this point.

Response. Thank you for raising this point. We conducted the visualization of pseudoknot in 1EHZ and visually showed that the number of layers (: pseudoknot order) is changed when you take non-canonical base pairs into account, which is described in the README.md. This example will help users with understanding the importance of non-canonical base pairs.

Effect of include_all (DSSR, 1EHZ)

Below is a concrete example showing how include_all changes the detected pseudoknot layers.

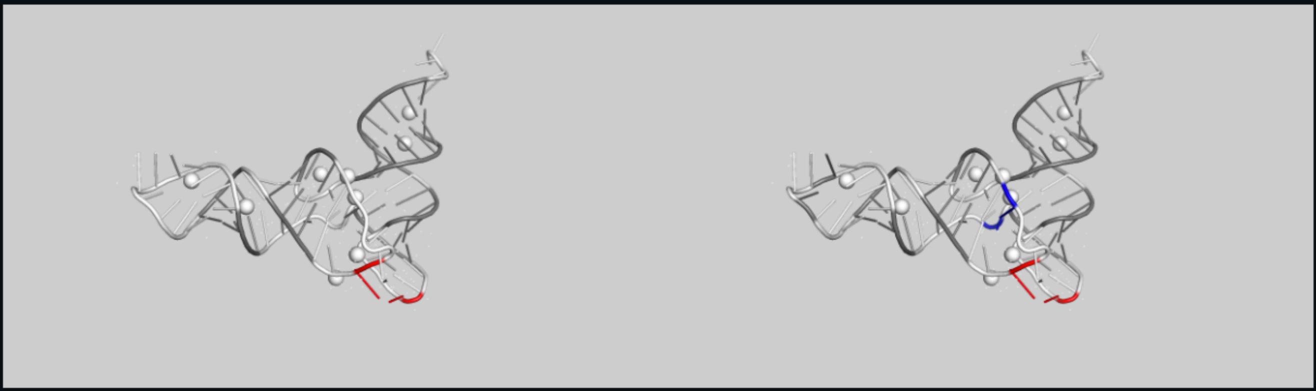

- Left: canonical only (annotator=DSSR)
- Right: include_all (annotator=DSSR)
- As visible, including non-canonical pairs increases the pseudoknot order (number of layers). As you can see, non-canonical pairs increase the pseudoknot order (number of layers).

> # Some specific points to consider

> - lines 24-25: "This software allows users to input RNA tertiary structure data in PDB format." PDB is a legacy format that is being phased out, so support for the mmCIF format should be added (or mentioned explicitly).

Response. Thank you for raising this point. Our software has **originally supported mmCIF**, however, it was not stated in the previous manuscript. We now state it explicitly in the manuscript (p.2 l.37–39, p.2 l.49–p.3 l.52, Fig1 caption, p.3 l.69 and p.4 l.82 and README.md).

To address this need, we developed the PseudoknotVisualizer, a tool that visualizes pseudoknots within RNA tertiary structures. This software allows users to input RNA tertiary structure data in the PDB or mmCIF format. It identifies pseudoknots and clarifies their spatial layouts in the widely used 3D molecular viewers, Chimera (12) and

> - *Fig 2 legend: "white represents unpaired bases;" --* ***White represents non-canonical base pairs or unpaired bases****. Again, having the different layers in separate PyMOL selections would facilitate further analysis.*

Response.
Thank you for pointing this out. We have re-rendered Fig. 2 with annotator=RNAView and include_all enabled, and we updated the legend accordingly. **In the revised figure, white denotes unpaired bases only. Gray indicates paired bases in the core layer** (layer 0), **including non-canonical base pairs**, and red/blue/green mark paired bases in pseudoknot layers 1/2/3, respectively. Thus, non-canonical pairs that fall in pseudoknot layers are colored by their layer (not white). The caption now states these settings for transparency (p.5 Fig2). As mentioned above, we implemented "PyMOL selections" for each layer (and a selection for unpaired nucleotides) to facilitate further analysis and export (p.3 l.62-64).

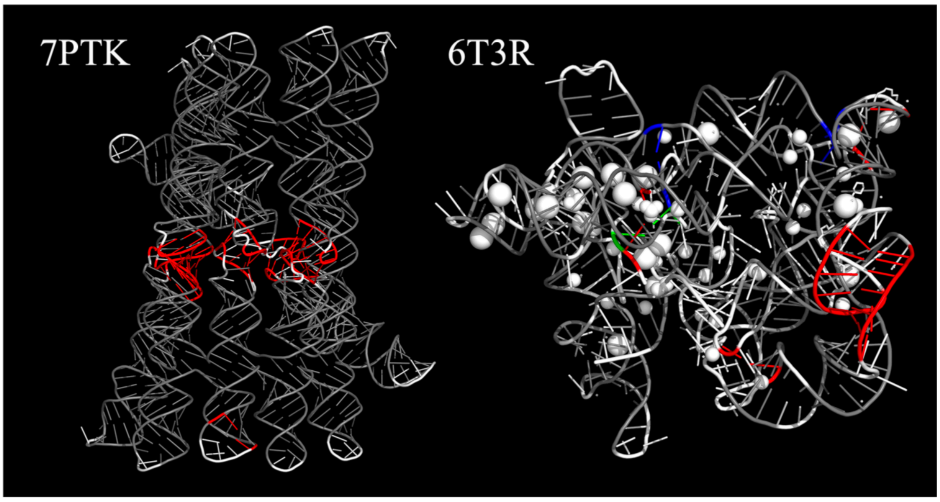

Fig 2. Examples of the pseudoknotted structures visualized by PseudoknotVisualizer are shown. This figure illustrates examples from PDB IDs 7PTK (20) and 6T3R (21), both of which are large sequences exceeding 300 nucleotides in length, making them particularly challenging to analyze visually without computational assistance. Note that, as demonstrated in figure 1, all structural elements were set to white prior to applying the PseudoknotVisualizer coloring operation for clarity. These visualizations were generated with `annotator=RNAView` and the option `include_all`, thereby including non-canonical pseudoknots. The color scheme is as follows: white represents unpaired bases; gray indicates paired bases including non-canonical base pairs in the core layer; red, blue, and green represent paired bases in pseudoknot layers 1, 2, and 3, respectively.

> - *line 72: "Using the pseudoknot layer decomposition algorithm described in Section 2" --section numbering error. The "Main Features" section is not numbered, and current section "Statistics on Pseudoknots" should be Section 3 (instead of 2)*

Response. Thank you for pointing this out. We have corrected the **section numbering** and the **in-text cross-reference**. The description of the pseudoknot-layer decomposition algorithm is now located in a properly numbered section, and the sentence in question now points to that section to ensure accurate navigation.

2 Design and Implementation

46

> - line 78: "a dataset of 1,788 RNA chains" -- Please specify these RNA chains long with their PDB IDs in the supplementary materials or the GitHub repository.

Response. Thank you for the suggestion. We re-ran the study using **RNAsolo (v2)** as our primary source and now provide a complete, machine-readable list of all RNA chains used (PDB ID + chain identifier). **The dataset list is deposited in the repository at analysis/dataset/pdbid_chains.txt**. We also record the **retrieval date (July 25, 2025)** and the filters applied (RNA-only; resolution ≤ 4.0 Å) (p.4 l.109-113). In addition, we have added access dates for the PDB links cited in the Introduction (p.2 l.15-19), and we report the **annotator versions** (RNAView, DSSR) in the Supplementary Information (Supplementary S1).

in several RNA viruses (3; 4; 5). Due to the labor-intensive process of determining RNA tertiary structures, the number of RNA structures recorded in databases is only about 3.80 % compared to that of proteins (`https://www.rcsb.org/stats/explore/polymer_entity_type`, access date: 2025/08/31). This gap underscores the need for highly accurate RNA tertiary structure

Using the pseudoknot layer decomposition algorithm described in Section 2, we decomposed 1,915 chains from RNAsolo (22) into pseudoknot-free layers (filters: repository=BGSU, redundancy=all class members, molecule=solo RNA, method=all, resolution $\leq$ 4.0, Å; accessed 2025-07-25). RNAsolo provides pure RNA structures, deriving from Protein Data Bank. Canonical base pairs were defined as Watson–Crick [illegible]

S1. Annotators

DSSR `v1.9.10-2020apr23`; RNAView `v2.0.0 (Jan 2024)`.
