# Supplementary material for: PseudoknotVisualizer: Visualization of pseudoknots on three-dimensional RNA structures
Source: PLoS Comput Biol. 2025 Nov 20;21(11):e1013693. doi: 10.1371/journal.pcbi.1013693 (PMC12654949; doi:10.1371/journal.pcbi.1013693)
Supplement: Attachment — Submitted filename: Response_to_Reviewers.pdf [file pcbi.1013693.s007.pdf]

Response to Reviewers

(PCOMPBIOL-D-25-01111R1, Minor Revision)

Reviewer 1

> *I appreciate that the authors positively responded to all my comments. I am aware that it required quite a lot of effort but the text is much more clear now. I do not have any other questions/comments.*

Response: Thank you very much for your positive assessment. Your earlier comments motivated us to conduct analyses again, which strengthened the reliability of our results, and clarified how the choice of base-pair annotators and decomposition algorithms can affect outcomes.

Reviewer 2

> *This new version has made some clarifications, but they still insist on a redefinition of the term pseudoknot that is absolutely unnecessary and conflicts with the actual definition of pseudoknot (the arrangement of two helices of WC base pairs where one of the helices involves residues in the loop of the other helix, [Batey, Rmabo, Doubna, 1999]), and ignores all the literature on other RNA 3D architectures in the RNA loops involving nonWC base pairs (Westhof, Leontis).*

Response: We sincerely apologize for our inappropriate response. In the previous revision, we redefined unnecessary "pseudoknot", since we had misunderstood your intention ("*Pseudoknots are a particular subset of non-tested interactions that involve Watson-Crick base pairing only. The rest of the non-nested interactions involve non-WC base pairing*") in the first review comment. In correspondence with your intention, we have now realigned our terminology with proper usage; we use "pseudoknot" for the non-nested interactions formed only by canonical base pairs (Watson-Crick base pairs and Wobble base pairs), in keeping with the classical definition [Batey, Rambo, Doubna, 1999]. In addition, the rest of non-nested interactions mediated by non-canonical pairs are referred to as "non-nested interactions involving non-canonical base pairs" throughout the revised manuscript.

Accordingly, when non-canonical base pairs are included for visualization ("all pairs"), we refer to the resulting layers as "non-nested layers," and we reserve "pseudoknot order" exclusively for the canonical-only setting. We also added citations to Batey, Rambo, and Doudna (1999) for the definition of a pseudoknot and to the Leontis–Westhof classification (2001) for non-canonical base pairs so that our terminology follows established usage. With these changes, terminology and notation are consistent across the manuscript. Thank you very much for prompting this important clarification.

Below are the detailed changes made in this version.

1. Author Summary. Instances labeled "pseudoknot layer" actually referred to layers obtained when non-canonical base pairs were included in the layer decomposition. To avoid mislabeling, we now describe them as non-nested layers.

> our tool to 1,915 RNA chains from the PDB revealed that canonical base pairs predominate in the core layer (87 %), while non-canonical base pairs are enriched (30 %) in the other non-nested layers; most chains exhibit no more than three layers of pseudoknots. By clarifying pseudoknot architecture in RNA 3D structures, PseudoknotVisualizer accelerates structural analysis and supports the development of computational structure prediction methods.

2. Introduction. We clarify that "pseudoknot" is the subset of non-nested interactions formed only by canonical base pairs (Watson-Crick + GU wobble), following the wording used by Reviewer 2 in the previous round. The rest of the non-nested interactions involve non-canonical base pairs. For reference, the reviewer's phrasing in the previous revision was: "Pseudoknots are a particular subset of non-[nested] interactions that involve Watson–Crick base pairing only. The rest of the non-nested interactions involve non-WC base pairing."

> Pseudoknots are structural motifs formed by base pairing between two single-stranded regions separated by an intervening stem; their base pairs are non-nested interactions (they cross when drawn in two dimensions) (1; 2). In many contexts, the term "pseudoknot" is the subset of the non-nested interactions involving only canonical base pairs (Watson–Crick (3) and wobble (4)). The rest of the non-nested interactions involve non-canonical base pairs (5; 6; 7) . These non-nested interactions, including pseudoknots, are integral to various RNA tertiary formations and play biologically

3. We state explicitly that the software identifies pseudoknots (canonical-only) and other non-nested interactions (non-canonical-mediated).

> To address this need, we developed the PseudoknotVisualizer, a tool that visualizes pseudoknots within RNA tertiary structures. This software allows users to input RNA tertiary structure data in the PDB or mmCIF format. It identifies pseudoknots (and other non-nested interactions involving non-canonical base pairs) and clarifies their

4. The decomposition algorithm applies to both pseudoknots and other non-nested interactions. When non-canonical base pairs are included, we refer to the resulting layers (excluding the core layer) as "non-nested layers," not "pseudoknot layers."

> could yield different results. We can also extend this algorithm to all non-nested interactions, including pseudoknots; in that case, we refer to the resulting layers as non-nested layers rather than pseudoknot layers because pseudoknot is used only for the non-nested interactions involving only canonical base pairs (see Introduction).

5. Because analyses that include non-canonical pairs fall outside the scope of "pseudoknot", the results labeled "all pairs" are consistently reported as "non-nested layers."

> were: RNAView (canonical only) 1,726 chains, of which 703 had at least one pseudoknot layer; RNAView (all pairs) 1,789 chains, of which 756 had at least one non-nested layer; DSSR (canonical only) 1,718 chains, of which 687 had at least one pseudoknot layer; DSSR (all pairs) 1,727 chains, of which 788 had at least one non-nested layer. Using

6. In Figure 2, the visualization includes non-canonical pairs; therefore, all instances of "pseudoknot" were replaced with "non-nested interactions," and "pseudoknot layer(s)" with "non-nested layer(s)."

> **Fig 2.** Examples of the structures which has non-nested interactions involving canonical base pairs and non-canonical base pairs visualized by PseudoknotVisualizer are shown. This figure illustrates examples from PDB IDs 7PTK (27) and 6T3R (28), both of which are large sequences exceeding 300 nucleotides in length, making them particularly challenging to analyze visually without computational assistance. Note that, as demonstrated in figure 1, all structural elements were set to white prior to applying the PseudoknotVisualizer coloring operation for clarity. These visualizations were generated with `annotator=RNAView` and the option `include_all`, thereby including the non-nested interactions involving non-canonical base pairs. The color scheme is as follows: white represents unpaired bases; gray indicates paired bases including non-canonical base pairs in the core layer; red, blue, and green represent paired bases in non-nested layers 1, 2, and 3, respectively.

7. We replaced "pseudoknot order" with "number of layers." We also note that when only canonical pairs are considered, this corresponds to pseudoknot order.

> Figure 3a shows the distribution of the number of non-nested layers (when considering only canonical base pairs, this corresponds to the pseudoknot order), annotating with DSSR. With canonical pairs only, most chains have ≤ 3 layers;

8. For the all-base-pairs case (including non-canonical pairs), we use "non-nested layer(s)" consistently (figure labels and captions aligned).

> We then extracted chains which have at least one non-nested layer. Figure 3(b) summarizes, for each layer, the per-chain fraction of non-canonical versus canonical pairs. The layer0 (core layer) is dominated by canonical pairs, whereas non-nested layers tend to show a higher fraction of non-canonical pairs. RNAView analysis again

9. Since our study investigates the base pairs contained in pseudoknot layers or non-nested layers, we explicitly use the term "non-nested layer(s)" where appropriate; for readability we also simplified phrases such as "different pseudoknot layers" to "different layers."

> ## 4 Availability and Future Directions
>
> Our present work provides an overview of base pairs which form the non-nested interactions, including pseudoknots. Our next step is to conduct a comprehensive statistical analysis of these interactions to better understand RNA structure. Specifically, we will quantify the distribution of the types of base-pairs which form the non-nested interactions and structural contexts and assess their contributions to 3D structure folding. Insights from these analyses will guide updates to

10. Figure 3 now presents paired views (canonical-only vs. all pairs), with x-labels and captions reflecting the canonical/non-canonical distinction (e.g., “non-nested layers” in panel (b)). Supplementary Fig. S1 was updated in the same way.

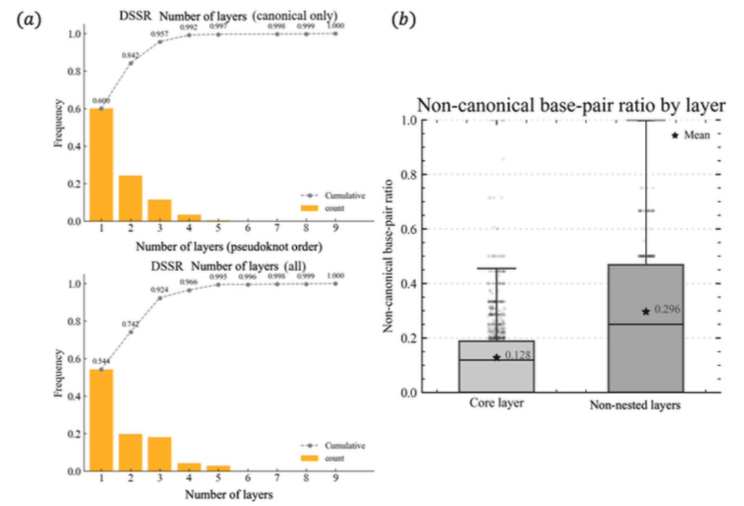

Fig 3. (a) Both graphs represent the frequency of the number of layers in RNA chains from PDB entries on the condition that these entries contain at least one base pair. The top graph considers only canonical base pairs, showing that over 80 % of chains have at most 2 layers. The bottom graph includes all base pairs, including non-canonical ones, demonstrating larger number of the layers compared to the top graph. Nevertheless, over 80 % of chains still have at most 3 layers. (b) Distribution of canonical and non-canonical base pairs across different decomposed layers in RNA molecules that contain at least one pseudoknot or other non-nested interactions, as determined by the PseudoknotVisualizer. Results shown were obtained using DSSR as the base-pair annotator.

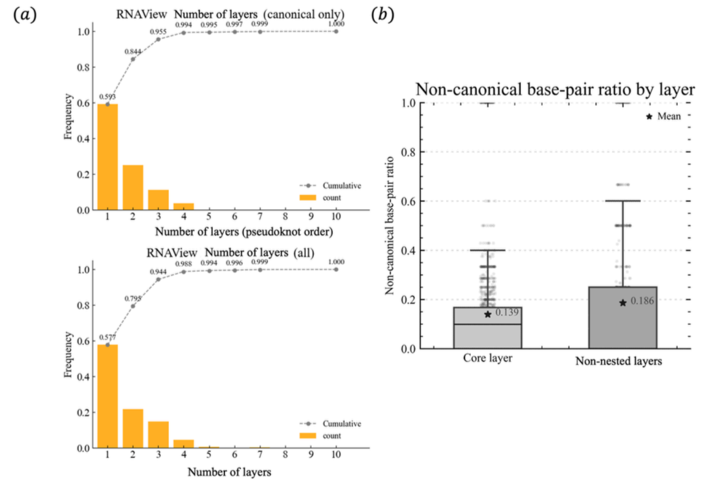

Figure S1: **Supplementary analyses.** (a) Distribution of the number of layers when using RNAView as the annotator. Consistent with the DSSR-based analysis in Fig. 3(a), including non-canonical base pairs in the decomposition increases the number of layers compared with using canonical pairs only. (b) Distribution of canonical and non-canonical base pairs across different decomposed layers in RNA molecules that contain at least one pseudoknot or other non-nested interactions. Similar to Fig. 3(b), non-nested layers exhibit a higher fraction of non-canonical base pairs than the core layer.

Reviewer 3

The authors have addressed my comments and concerns.

Response: We thank the reviewer for the positive assessment. In particular, your practical, implementation-oriented suggestions were invaluable. They guided several refinements that improved the software's robustness, error handling, and user experience. We are grateful for your contribution to improving the tool.

Obvious typographical correction. We removed the stray phrase "pseudoknot-free," which was unnecessary in context. This is a purely editorial fix and does not affect any analyses, results, or conclusions. We trust that this correction is acceptable.

before

> > 4 remain rare. The observed distribution of pseudoknot-free pseudoknot orders is consistent with findings from previous studies (8), and RNAView results show the same

after

> including non-canonical pairs slightly shifts the tail, but orders > 4 remain rare. The observed distribution of pseudoknot orders is consistent with findings from previous
